# Effect of the French Oak Wood Extract Robuvit on Markers of Oxidative Stress and Activity of Antioxidant Enzymes in Healthy Volunteers: A Pilot Study

**DOI:** 10.1155/2014/639868

**Published:** 2014-08-31

**Authors:** Martina Horvathova, Zuzana Orszaghova, Lucia Laubertova, Magdalena Vavakova, Peter Sabaka, Peter Rohdewald, Zdenka Durackova, Jana Muchova

**Affiliations:** ^1^Institute of Medical Chemistry, Biochemistry and Clinical Biochemistry, Faculty of Medicine, Comenius University, Sasinkova 2, 811 08 Bratislava, Slovakia; ^2^Institute of Medical Biochemistry, Jessenius Faculty of Medicine, Comenius University, Malá Hora 4, 036 01 Martin, Slovakia; ^3^2nd Department of Internal Medicine, Faculty of Medicine, Comenius University, Mickiewiczova 13, 813 69 Bratislava, Slovakia; ^4^Westfälische Wilhelms-Universität Münster, Institut für Pharmazeutische Chemie, Twenteweg 15, 48161 Münster, Germany

## Abstract

We examined *in vitro* antioxidant capacity of polyphenolic extract obtained from the wood of oak *Quercus robur* (QR), Robuvit, using TEAC (Trolox equivalent antioxidant capacity) method and the effect of its intake on markers of oxidative stress, activity of antioxidant enzymes, and total antioxidant capacity in plasma of 20 healthy volunteers. Markers of oxidative damage to proteins, DNA, and lipids and activities of Cu/Zn-superoxide dismutase (SOD), catalase (CAT), and glutathione peroxidase (GPx) were determined in the erythrocytes. We have found an *in vitro* antioxidant capacity of Robuvit of 6.37 micromole Trolox equivalent/mg of Robuvit. One month intake of Robuvit in daily dose of 300 mg has significantly decreased the serum level of advanced oxidation protein products (AOPP) and lipid peroxides (LP). Significantly increased activities of SOD and CAT as well as total antioxidant capacity of plasma after one month intake of Robuvit have been shown. In conclusion, we have demonstrated for the first time that the intake of Robuvit is associated with decrease of markers of oxidative stress and increase of activity of antioxidant enzymes and total antioxidant capacity of plasma *in vivo*.

## 1. Introduction

The French oak wood extract Robuvit (Horpag Research Ltd.) is a registered proprietary water extract obtained from the wood of* Quercus robur* (QR). The plant belongs to the plant family Fagacae, genus* Quercus*.

The oak wood used for Robuvit originates exclusively from oak trees grown in France. Oak wood contains a specific profile of tannins named roburins that are part of the ellagitannins (ETs). Robuvit is standardized and specified to contain at least 20% of roburins (A, B, C, D, and E) including grandinin. The two most abundant ETs in the Robuvit are stereoisomers vescalagin and castalagin, which were originally isolated and described by Mayer et al. [1]. Roburins and grandinin are dimers of these compounds or differ by the presence of a pentose substituent. They were isolated and identified later by du Penhoat et al. [2]. Further to the roburins, Robuvit contains monomeric vescalagin and castalagin as well as ellagic acid (EA) and gallic acid (GA) [3].

Owing to their unique molecular structure roburins are very potent antioxidants. Humans have been exposed to these compounds for centuries from wine and spirits that matured in oak wood barrels. Oak wood is currently the only known food source of roburins, and, according to this specificity, the major source of roburins in human diets results from the consumption of wine and spirits (cognac and whiskey) traditionally matured, aged, and stored in oak barrels [4].

Little is known about roburins bioavailability and biological effects. Natella et al. [3] found out that Robuvit is bioavailable to humans and its consumption is associated with increase of antioxidant capacity at hydrophilic conditions. They identified beside GA and EA also metabolites of ETs named urolithins in the plasma of volunteers after intake of Robuvit. Urolithins are metabolites of EA, which are released from ETs by hydrolysis in the intestine by gut microflora [5]. Effects of EA, one of compounds of Robuvit, were examined in many models* in vitro* and* in vivo*. EA is characterized by antioxidant [6–9], anticarcinogenic [10–13], antiproliferative [9, 14], anti-inflammatory [15, 16], proapoptotic [17], and antiplatelet properties [18].

The aim of our study was to examine* in vitro* antioxidant capacity of Robuvit using TEAC method and the* in vivo* effect of its intake on oxidative stress in healthy volunteers. The level of markers of oxidative stress (markers of oxidative damage to proteins, lipids, and DNA), activities of antioxidant enzymes Cu/Zn-superoxide dismutase (SOD), catalase (CAT), and glutathione peroxidase (GPx) and total antioxidant capacity of plasma were investigated.

## 2. Material and Methods

### 2.1. Subjects

Twenty healthy volunteers (12 women and 8 men, mean age 54.2 ± 6.56; range: 45–65 years) were included in the study. All participants gave a written informed agreement to participate in the study. The study was approved by the Ethical Committee of University Hospital and Faculty of Medicine, Comenius University in Bratislava, Slovakia. Subjects with acute inflammatory, renal and cardiovascular diseases, and diabetes mellitus and women with hormone substitution therapy were excluded from the study.

The 8-week experiment was divided into three periods. In the first period (run-in period) volunteers were instructed to control their diet for 2 weeks. No additional antioxidants like vitamins C, E or coenzyme Q or excess of chocolate, red wine, or beer should be consumed. Drinking a cup of green tea, 2 dL red wine, or 1 beer daily was allowed. After run-in period, Robuvit administration began (week 0). Volunteers took 1 capsule (100 mg) of extract Robuvit three times a day (300 mg daily) for 4 weeks, followed by two-week wash-out period in which capsules were not administered. The extract was supplied by Horphag Research Ltd., Switzerland.

### 2.2. Sample Preparation

The venous blood was collected after 12-hour overnight fast into commercial serum tubes and EDTA coated tubes. Within 1 hour of collection, blood was centrifuged (700 ×g, 5 min); serum and plasma were aliquoted and frozen at −80°C until analysis. For isolation of erythrocytes, blood was washed three times with 0.15 mol/L NaCl solution. After final centrifugation (700 ×g, 7 min), erythrocytes were haemolysed by addition of triple volume of distilled water and haemolysate was frozen at −20°C until analysis.

The samples of blood were taken after run-in period (week 0), at the end of intervention period (week 4) and at the end of wash-out period (week 6).

In the serum of the basic biochemical parameters, the concentrations of advanced oxidation protein products (AOPP) and lipid peroxides (LP) were determined; in the plasma the levels of advanced glycation end-products (AGEs), 8-isoprostanes (8-isoP), protein carbonyls, and total antioxidant capacity were examined. In isolated lymphocytes marker of oxidative damage to DNA and 8-oxoguanin (8-oxoG) was assessed. The concentration of haemoglobin by Drabkin method and SOD, CAT, and GPx activities were measured in haemolysate of erythrocytes.

### 2.3. Chemicals

ABTS [2,2′-azinobis-(3-ethylbenzothiazoline-6-sulfonic acid) diammonium salt], Trolox (6-hydroxy-2,5,7,8-tetramethylchroman-2-carboxylic acid), dextran sulphate, chloramine T, igepal, o-phenylenediamine, guanidine hydrochloride, Tween 20, bovine serum albumin (BSA), histopaque 1083, low melting point (LMP) agarose, normal melting point (NMP) agarose, Triton X-100, hydrogen peroxide, AAPH [2,2′-azobis (2-amidinopropane) dihydrochloride], and formamidopyrimidine-DNA glycosylase (Fpg) were purchased from Sigma-Aldrich (USA); sodium azide and DAPI (4,6-diamidino-2-phenylindole dihydrochloride) were purchased from Merck (Germany); TPTZ (2,4,6-tripyridyl-s-triazine), disodium fluorescein, 2,4-dinitrophenylhydrazine (DNPH), and glycerol were purchased from Fluka (Germany); all other chemicals were purchased from Lachema (Czech republic); Robuvit was obtained from Horphag Research Ltd. (Geneva, Switzerland).

### 2.4. Measurement of Basic Biochemical Parameters

Basic biochemical parameters (bilirubin, glucose, gamma-glutamyl transferase (GMT), alkaline phosphatase (ALP), aspartate aminotransferase (AST), alanine aminotransferase (ALT), uric acid (UA), total proteins (TP), total cholesterol (TCh), triacylglycerols (TAG), HDL-cholesterol (HDL-chol), LDL-cholesterol (LDL-chol), and VLDL-cholesterol (VLDL-chol)) were determined at an accredited clinical biochemistry and haematology laboratory using a Hitachi 911 analyzer by Roche diagnostics kits (Switzerland).

### 2.5. Measurement of* In Vitro* Antioxidant Capacity of Robuvit—TEAC (Trolox Equivalent Antioxidant Capacity) Assay

57.8 mg of Robuvit was dissolved in 10 mL of distilled water, vortexed for 5 min, centrifuged (3000 ×g/10 min), and diluted to concentrations of 10^−4^ to 10^−9^ g/mL with distilled water.

*In vitro* antioxidant capacity of Robuvit was measured by method according to Re et al. [19]. It is a decolourization assay for measurement of both lipophilic and hydrophilic antioxidants. The radical monocation of 2,2′-azinobis-(3-ethylbenzothiazoline-6-sulfonic acid) diammonium salt (ABTS^•+^) is generated by oxidation of ABTS with potassium persulfate and is reduced in the presence of hydrogen-donating antioxidants into colourless form. Quantification was performed using the dose-response curve for reference antioxidant Trolox water soluble form of vitamin E. Robuvit antioxidant capacity was calculated as Trolox equivalents per mg of Robuvit.

### 2.6. Determination of Advanced Oxidation Protein Products (AOPP)

Serum concentration of AOPP was measured by modified method according to Witko-Sarsat et al. [20]. 200 *μ*L of blood serum diluted 1 : 5 with phosphate buffer saline (PBS, pH 7.4), 200 *μ*L of chloramine T (0–100 *μ*mol/L) for calibration, and 200 *μ*L of PBS as blank were applied on a microtiter plate. 10 *μ*L of 1.16 mol/L potassium iodide was added to standards and 20 *μ*L of acetic acid was added into all samples. Absorbance at 340 nm was measured and results are presented in *μ*mol/L.

### 2.7. Determination of Protein Carbonyls

The concentration of protein carbonyls in plasma was measured by ELISA method according to Buss et al. [21]. Briefly, carbonyls of sample were derivatized by 2,4-dinitrophenylhydrazine (DNPH). A biotin-conjugated rabbit IgG raised against DNPH was used as a primary antibody and a monoclonal goat anti-rabbit IgG antibody labelled by horseradish peroxidase (HRP) as a secondary antibody. Product of enzymatic reaction catalyzed by HRP has absorption maximum at 490 nm. Results were calculated from calibration curve using oxidized BSA as a standard and expressed in ng/mL.

### 2.8. Determination of Advanced Glycation End-Products (AGEs)

The concentration of plasma AGEs was determined by modified method according to Kalousová et al. [22]. Determination was based on measurement of total fluorescence at wavelengths (exc./emis.) 345 nm/465 nm in the plasma diluted with 0.01 mol/L PBS. Results are expressed in AU/g of proteins.

### 2.9. Determination of Oxidative DNA Damage by Comet Assay

Oxidative DNA damage in lymphocytes was evaluated by the enzymatically modified comet assay using formamidopyrimidine DNA glycosylase (Fpg) recognizing oxidized purines [23]. Lymphocytes were isolated using Histopaque 1083 according to the manufacturer's protocol. Oxidative DNA damage was expressed as a number of 8-oxoG per 10^6^ guanines according to ESCODD [24].

### 2.10. Determination of 8-Isoprostanes (8-isoP)

8-isoP were determined by the commercial EIA kit (Cayman Chemical Company, USA). This assay is based on the competition between 8-isoP and an 8-isoprostane-acetylcholinesterase (AChE) conjugate (8-isoprostane Tracer) for a limited number of 8-isoprostane-specific rabbit antiserum binding sites. Because the concentration of the 8-isoprostane Tracer is held constant while the concentration of 8-isoP varies, the amount of 8-isoprostanes Tracer that is able to bind to the rabbit antiserum will be inversely proportional to the concentration of 8-isoP in the well. The plate is washed to remove any unbound reagents and then Ellman's reagent (which contains the substrate to AchE) is added to the well. The product of enzymatic reaction has a distinct yellow colour and absorbs the light at 405 nm. The concentration of 8-isoP is expressed in pg/mL.

### 2.11. Determination of Lipid Peroxides (LP)

Concentration of LP in serum was measured by method according to El-Saadani et al. [25]. The analysis was based on the ability of LP to convert iodide to iodine, which can be measured spectrophotometrically at 365 nm. Results are presented in nmol/mL of plasma.

### 2.12. Measurement of Activity of Cu/Zn-Superoxide Dismutase (SOD)

Activity of SOD was determined by the commercial kit number 19160 (Fluka, Germany), using bovine Cu/Zn-SOD as a standard (Sigma, Germany). Superoxide radical is generated from xanthine and oxygen by xanthine oxidase. Superoxide radical reduces Dojindo's highly water-soluble tetrazolium salt, WST-1 (2-(4-iodophenyl)-3-(4-nitrophenyl)-5-(2,4-disulfophenyl)-2H-tetrazolium, monosodium salt) to form a yellow water-soluble formazan dye with maximum absorption at 450 nm. At the presence of SOD the reduction of WST-1 is decreased. 1 U of SOD activity is defined as the enzyme activity causing 50% inhibition of reduction of WST-1. SOD activity is expressed in U SOD/mg Hb.

### 2.13. Measurement of Activity of Catalase (CAT)

Catalase activity was determined by modified method of Bergmeyer [26]. 250 *μ*L of H_2_O_2_ was added into 50 mL phosphate buffer saline (PBS, 50 mmol/L, pH = 7.0) and formed mixture was diluted to get absorbance 0.7–1.0. 30 *μ*L of sample was added into 2 mL of PBS + H_2_O_2_ mixture. Change of absorbance at wavelength 240 nm was measured for 1 min. Distilled water was used as blank. Activity of catalase is expressed in *μ*kat/g Hb.

### 2.14. Measurement of Activity of Glutathione Peroxidase (GPx)

Activity of GPx was determined by the commercial kit (Sigma-Aldrich, USA). This kit uses an indirect determination method. It is based on the oxidation of glutathione (GSH) to oxidized glutathione (GSSG) catalyzed by GPx, which is then coupled to the recycling of GSSG back to GSH utilizing glutathione reductase and NADPH. The decrease in NADPH absorbance measured at 340 nm during the oxidation of NADPH to NADP^+^ is indicative of GPx activity. Activity of GPx is expressed in U/g Hb.

### 2.15. Measurement of Total Antioxidant Capacity of Plasma—ORAC (Oxygen Radical Absorbance Capacity) Assay

The ORAC assay was measured by modified method according to Huang et al. [27]. The method is based on the oxygen radical absorbance capacity where a peroxyl radical (AAPH) oxidizes fluorescein in competition with an antioxidant (in the plasma). AAPH leads to production of ROO^−^ that reacts with the fluorescein. This results in a gradual loss of fluorescence intensity. Antioxidants, which are present in the plasma, inhibit the reaction between ROO^−^ and the fluorescent probe, which slows down or inhibits the fluorescence degradation. The ORAC assay indicates the antioxidant capacity of the plasma by using the area under the curve in combination with inhibition time and inhibition potency. Results are presented as mmol of Trolox/L.

### 2.16. Measurement of Total Antioxidant Capacity—FRAP (Ferric Reducing Ability of Plasma) Assay

The FRAP was determined by the method according to Benzie and Strain [28]. The method is based on the reduction of Fe^3+^-TPTZ complex to the ferrous form at low pH (pH 3.6). This reduction is monitored spectrophotometrically at 593 nm. Quantification was performed using the dose-response curve for reference antioxidant Trolox. Results are presented as mmol of Trolox/mL.

### 2.17. Statistical Analysis

Results are presented as a mean ± standard deviation (SD) or median and interquartile range (IQ, 1st quartile, 3rd quartile). Parametric Student's paired *t*-test was used for statistical analysis of data with a Gaussian distribution and nonparametric Mann-Whitney *U*-test or Wilcoxon's signed ranks test was used for data with a non-Gaussian distribution. Value *P* < 0.05 was considered statistically significant. Statistical software StatsDirect 2.3.7. (StatsDirect Sales, Sale, Cheshire M33 3UY, UK) was used.

## 3. Results

Administration of Robuvit for 4 weeks decreased weight of volunteers, but because of high interindividual variability of body weight this change (−0.7 kg) has no relevance. Values of BMI were not significantly changed after week 4 and week 6.

Basic biochemical parameters were investigated in fasting venous blood in examination periods 0, 4, and 6: bilirubin, glucose, GMT, ALP, AST, ALT, UA, TP, TCh, TAG, HDL-chol, LDL-chol, and VLDL-chol. All values of mentioned biochemical parameters were in the physiological ranges.

We did not observe serious unwanted effects of Robuvit administration.

*In vitro* antioxidant capacity of Robuvit was calculated as 6.37 micromole Trolox equivalent/mg of Robuvit.

Concentrations of markers of oxidative damage to proteins (AOPP, protein carbonyls, and AGEs), DNA (8-oxoG), and lipids (8-isoP and LP), activities of antioxidant enzymes (SOD, CAT, and GPx), and total antioxidant capacity (ORAC and FRAP) are summarized in Table 1. Four weeks administration of Robuvit significantly decreased the concentration of AOPP in serum by 42.09% and LP by 21.53%. This effect persisted after week 6 where the level of AOPP remained decreased by 42.85% and LP by 19.79%.

The concentrations of protein carbonyls, AGEs, 8-oxoG, and 8-isoP were not significantly changed after week 4. Only after week 6 we found out significantly decreased level of protein carbonyls by 7.77% in comparison to week 0.

We measured activities of SOD, CAT, and GPx in haemolysate of erythrocytes. Administration of Robuvit significantly increased activity of SOD by 11.76% and this effect persisted after week 6 where the activity of SOD was even more increased by 13.22%. We also found a significantly higher activity of CAT after week 4 by 15.54%. Activity of GPx was not influenced by intake of Robuvit.

Total antioxidant capacity of plasma was determined by two methods with different mechanism of action: ORAC and FRAP. We did not find significant changes in ORAC values in weeks 4 and 6 in comparison to week 0. However, we found out significantly increased level of FRAP values by 6.31% after week 4.

## 4. Discussion

We have studied* in vitro* antioxidant capacity of the French oak wood extract Robuvit and the* in vivo* effect of its administration on markers of oxidative damage to biomolecules, activity of antioxidant enzymes, and total antioxidant capacity in 20 healthy volunteers. Our results represent the first investigation of extract from the wood of* Quercus robur*, Robuvit on oxidative stress in humans.

We have determined* in vitro* antioxidant capacity of Robuvit by TEAC method. Natella et al. [3] determined the antioxidant capacity of Robuvit by ORAC assay as 648 nmol Trolox equivalents per mg extract in Robuvit, 10 times lower than our result (6370 nmol Trolox equivalents per mg), which can be caused by different method used or rather by different way of evaluation.

In our human trial we have found that Robuvit was able to protect significantly proteins and lipids against oxidation as we found significantly decreased levels of AOPP and LP in serum.

In addition, 4-week intake of Robuvit significantly increased the intracellular defence against oxidative stress by enhancing the activities of SOD and CAT in erythrocytes. We also found significantly increased FRAP values of total antioxidant capacity in the plasma (by 6.3%) after week 4 in healthy volunteers, but this small change may not have a biological relevance. Also Natella et al. [3] observed significantly increased total antioxidant capacity in the hydrophilic component of plasma (by 5.55%) measured by ORAC assay.

The mechanism for the antioxidant effect of polyphenols in Robuvit* in vivo* is most probably based on the antioxidant and biomodulating activity of the EA and metabolites of the roburins, the urolithins.

Following consumption of the oak wood extract Robuvit, besides EA and GA, three different urolithins were identified in plasma of volunteers [3]. Antioxidant effects of EA and urolithins were examined in many models* in vivo* and* in vitro*. They were associated with reduction of intracellular reactive oxygen species (ROS) and malondialdehyde (MDA) levels and increase of SOD activity in H_2_O_2_-treated T24 cells [9]. Similar results were observed also by Kim et al. [8] who found out that EA significantly reduced ROS level and MDA concentration in paraquat-induced A549 cells. Kavitha et al. [7] observed significant increased expression of antioxidant enzymes SOD, CAT, and GPx by EA in 7,12-dimethylbenz[a]anthracene- (DMBA-) induced hamster buccal pouch carcinogenesis model. Celik et al. [6] also found significantly increased activities of CAT and GPx in rat liver microsomes by EA treatment. Urolithins accumulate in different tissues of the body and they have been suggested to be responsible for biological effects observed as a consequence of the ingestion of ellagitannins-rich foods [29]. Our results show rather indirect antioxidant effects of Robuvit than direct ones. Although we observed significantly increased total antioxidant capacity, this change is too small to have important biological relevance. Robuvit increases antioxidant protection against oxidative damage to biomolecules probably through the stimulation of expression or activities of antioxidant enzymes, what is supported by increased activities of SOD and CAT after supplementation of Robuvit. However, precise mechanism of antioxidant effects is yet unknown and there are another studies needed.

## 5. Conclusions

In conclusion, we have confirmed for the first time that the intake of the French oak wood extract Robuvit is associated with a decreased damage to proteins and lipids, a stimulation of antioxidant enzymes (SOD and catalase), and a moderate increase of total antioxidant capacity of plasma in humans.

## Figures and Tables

**Table 1:** Markers of oxidative stress, activity of antioxidant enzymes, and total antioxidant capacity after weeks 0, 4, and 6.

Parameter	Week 0	Week 4	*P* (0 versus 4)	Week 6	*P* (0 versus 6)
AOPP (*μ*mol/L)	58.06 (49.32; 59.27)	33.62 (30.49; 35.56)	<0.0001	33.18 (28.44; 34.03)	<0.0001
PC (ng/mL)	0.296 ± 0.059	0.288 ± 0.052	n.s.	0.273 ± 0.054	0.0037
AGEs (AU/g prot.)	273.17 (249.01; 305.33)	296.17 (237.34; 323.05)	n.s.	286.07 (258.07; 322.95)	n.s.
8-oxoG/10^6^ G	0.61 (0.35; 0.91)	0.79 (0.47; 1.43)	n.s.	0.78 (0.38; 1.62)	n.s.
8-isoP (pg/mL)	24.59 (21.16; 33.77)	34.02 (24.16; 39.01)	n.s.	31.63 (23.98; 44.66)	n.s.
LP (nmol/mL)	42.55 (33.6; 61.54)	33.39 (28.17; 44.19)	0.0174	34.13 (28.10; 48.37)	0.0038
SOD (U/mg Hb)	42.42 ± 6.83	47.41 ± 9.93	0.0198	48.03 ± 5.27	0.0240
CAT (*μ*kat/g Hb)	5.60 (4.76; 6.12)	6.47 (6.11; 7.41)	0.0012	4.59 (3.72; 6.46)	n.s.
GPx (U/g Hb)	39.55 ± 9.16	40.74 ± 8.23	n.s.	36.21 ± 8.96	n.s.
ORAC (mmol/L)	8.97 (8.34; 9.61)	8.72 (8.03; 9.67)	n.s.	8.69 (8.08; 9.72)	n.s.
FRAP (mmol/mL)	0.586 ± 0.098	0.623 ± 0.115	0.0091	0.603 ± 0.124	n.s.

Data are presented as mean ± SD or median and IQ range (1st quartile; 3rd quartile); AOPP: advanced oxidation protein products; PC: protein carbonyls; AGEs: advanced glycation end-products; AU: arbitrary units; 8-isoP: 8-isoprostanes; LP: lipid peroxides; SOD: Cu/Zn-superoxide dismutase; CAT: catalase; GPx: glutathione peroxidase; ORAC: oxygen radical absorbance capacity; FRAP: ferric reducing ability of plasma; *P* < 0.05: significant; n.s.: not significant (*P* > 0.05).
